# Heterogeneity in public health service utilization and its relationship with social integration among older adult migrants in China: a latent class analysis

**DOI:** 10.3389/fpubh.2024.1413772

**Published:** 2024-08-07

**Authors:** Xiaolong Bu, Ting Wang, Qian Dong, Cuiping Liu

**Affiliations:** School of Nursing, Shandong First Medical University, Tai'an, China

**Keywords:** exploratory factor analysis, latent class analysis (LCA), older migrant population, social integration, utilization of basic public health services

## Abstract

**Background:**

The older adult migrant population in China is on the rise, which presents challenges for the national public health service system. However, the heterogeneity of public health service utilization and its relationship with social integration among the older adult migrant population remains unclear. This study aims to explore the heterogeneity the public health service utilization and how it relates to their social integration.

**Methods:**

A total of 6,178 older adult migrants from the China Migrants Dynamic Survey (CMDS) in 2017 were included in this study. Exploratory factor analysis was used to categorize social integration into four dimensions. Latent class analysis (LCA) was used to identify different sub-groups of public health service utilization. ANOVA and multivariate logistic regression were used to determine the characteristics of different sub-groups.

**Results:**

Three potential classes of public health service utilization were identified: low utilization of basic public health services class (*N* = 3,264,52.756%), medium utilization of basic public health services class (*N* = 1,743,28.172%), and high utilization of basic public health services class (*N* = 1,180,19.072%). Gender, education, extent of mobility, and move alone or not, flow time were all predictors of the class of public health service utilization. There were significant differences in social integration across potential categories (*p*<0.0001).

**Conclusion:**

The utilization of public health services of the older adult migrants is affected by many aspects. Social integration deserves attention as a significant influencing factor in the utilization of public health services. The government should pay attention to the characteristics of the older adult migrants and formulate relevant policies in a targeted manner in order to improve the utilization of public health services of the older adult migrants.

## 1 Introduction

As China progresses through industrialization and socialist modernization, the number of floating migrants is rapidly increasing. Recent data from the seventh national census indicates that the floating population in China has surpassed 370 million, indicating a 69.73% surge since the sixth national census in 2010 (1). The increasing floating population is crucial for the economic and social progress of the nation. With the rapid development of the economy and society, China is experiencing a rapid aging of its population and changes in demographic composition. As of the end of 2021, the population aged 60 and above had reached 267 million, representing 18.9% of the total population (1). Correspondingly, the number and proportion of older adult individuals within the floating population have also surged. According to data from the 2018 national dynamic monitoring of the floating population, older adult individuals comprised 39.7% of the floating population, including 60,000 individuals in the older age group (2). The increase in older adult migrants moving between regions has given rise to a range of societal challenges such as health issues (3), social isolation (4), concerns regarding psychological well-being (5, 6), and obstacles related to social welfare and integration (7). Research indicates that older adult members of the floating population are more susceptible to health problems and have a lower quality of life compared to younger migrants or local senior citizens (8, 9).

Providing public health services to its citizens has long been a persistent policy program in China (10). In 2009, China launched a new health reform endeavor, introducing the national basic public health service system (NBPHS) plan under the auspices of the health ministry (11). The primary objective of this initiative was to provide complimentary and discretionary basic health services, covering maternal and infant health, mental health management, older adult health management, and areas such as tuberculosis. Studies have underscored the significant impact of public health services on the health outcomes of older adult migrants. The government offers services such as physical examinations and health education to the floating population, facilitates access to medical care for the older adult floating population, and establishes health records through public health service projects. Research indicates that these interventions can positively influence the self-perceived health status and management of chronic conditions among the older adult floating population (12–15).

Various factors can influence the utilization of public health services among the older adult migrant population. Current research demonstrates that factors such as age, gender, and general demographic characteristics may differ among older adult migrants who access public health services (16, 17). Additionally, social integration can play a pivotal role. Social integration, a concept initially introduced by French scholar Émile Durkheim, posits that strong social integration can mitigate the incidence of suicide. Subsequently, scholars have further explored and refined this concept. Chinese scholar Yuan (18) defined social integration as the establishment of connections among diverse cultures, groups, and individuals based on mutual familiarity and adaptation. This process of establishing connections is crucial for promoting social cohesion. Concerning the social integration of the floating population, Sun (19) sees social integration as the process by which immigrants grow into local members and are empowered to participate in various social relationships. Given their limited adaptability and cognitive abilities (20), the challenge of social integration is particularly acute among older adult migrants compared to other cohorts. Effective integration can help older adult migrants in acclimating to their new environment, enhancing their involvement in local social activities, fostering better interpersonal communication, and ultimately elevating their quality of life and well-being. The struggle of older adult migrants to integrate seamlessly into their new surroundings can impede their access to public health services. However, the precise impact of social integration on the utilization of public health services among older adult migrants remains uncertain.

Limited research currently exists on the relationship between social integration and the utilization of public health services among migrants. Existing studies have primarily focused on demographic factors influencing the use of public health services. For instance, Yanwei Lin and colleagues examined public health service utilization among the older adult floating population, with a focus on age and medical insurance as key factors influencing their access to these services (21). Similarly, Dan Tang et al. explored the utilization of public health services among the older adult floating population, highlighting demographic factors such as household registration type, income level, and education in determining their engagement with these services (11). XueYao Wang investigated the impact of social integration on migrant populations’ access to health education and healthcare choices in a cross-sectional survey (22), representing the sole study known to explore the link between public health service utilization and social integration among migrants. Nevertheless, this research does not account for individual differences in service utilization or how the social integration of migrants across various age groups may influence their use of public health services. Other studies on public health service utilization by migrants have overlooked the heterogeneity within this population. Latent class analysis (LCA), a person-centered approach, can identify potential subgroups based on individual responses, thereby categorizing the population into several categories (23). LCA proves suitable for this study as the utilization of public health services among the older adult migrant population is inevitably heterogeneous due to the differences in educational level, household registration status, mobility patterns, and various other factors.

The objective of this study was to differentiate individual differences in the utilization of public health services among the older adult migrant population using LCA. Additionally, the study was also aimed to investigate how demographic characteristics and social integration impact their engagement with public health services.

## 2 Methods

### 2.1 Data source

The data used in this study was obtained from the 2017 China Migrants Dynamic Survey (CMDS), which employed a rigorous sampling method to gather information from 169,989 individuals within the floating population aged 15 and above. These individuals had been residing locally for at least 1 month and did not possess household registration in the area. According to the National Standard for New Basic Public Health Service (NBPHS) (third edition), the initial step to access public health services in one’s place of residence is to establish a health record. To fulfill this requirement, individuals must have resided in their current location for more than 6 months. Hence, this study specifically focused on mobile older adult individuals aged over 60 who met this residency criterion. After excluding non-compliant and invalid data, a total of 6,178 subjects were included in the analysis.

### 2.2 Variables

#### 2.2.1 Dependent variables

The National Health and Family Planning Commission launched a pilot program for equal access to NEPHS for migrants in 2013 and issued Guidance on the management of basic public health services for migrants in 2014. The guidelines identified six most important public health services for migrants as childhood vaccines, prevention and control of communicable diseases, maternal and child health, health records, family planning and health education (24). In the China Migrants Dynamic Survey, there was a section that focused on the utilization of public health services. Two aspects including ten items that are most pertinent to older individuals were chosen to evaluate their utilization of public health services in our study. These items have been previously validated in similar studies (11, 25). The first aspect concentrated on the establishment of health records and included one item. The second aspect pertained to the acceptance of health education and encompassed nine items: occupational diseases, tuberculosis, chronic diseases, prevention of sexually transmitted diseases, control of smoking, maternal and child health care, reproductive health, mental health, and emergency response to public emergencies. This ten items are binary variables that required a “yes” or “no” response. For the purpose of latent class analysis, we consider the “yes” option as “1” and the “no” option as “0”.

#### 2.2.2 Independent variables

The study focused on the social integration of the older adult floating population as the main independent variables. After conducting a thorough literature review and consulting with the research group, a set of nine items was selected to evaluate the social integration of this population (26). To facilitate subsequent analysis, an exploratory factor analysis was performed to process and analyze the data. The specific items and their corresponding scores are shown in Table 1.

**Table 1:** Evaluation indicators of social integration.

Item	Evaluation
What is your average monthly income in the past year?	0 ~ 1,999 = 1, 2,000 ~ 3,999 = 2, 4,000 ~ 5,999 = 3, Above the 6,000 = 4
What is your average monthly expenditure in the past year?	0 ~ 1,999 = 1, 2,000 ~ 3,999 = 2, 4,000 ~ 5,999 = 3, Above the 6,000 = 4
I like the city	1 = completely disagree; 2 = disagree; 3 = basic agree; 4 = complete agree
I noticed the changes in this city	1 = completely disagree; 2 = disagree; 3 = basic agree; 4 = complete agree
I would like to integrate into the local people and become one of them	1 = completely disagree; 2 = disagree; 3 = basic agree; 4 = complete agree
Since 2016, have you made advice or supervised the unit / community / village management	1 = no; 2 = occasionally; 3 = sometimes; 4 = often
Since 2016, have you reported the situation to relevant government departments in various ways	1 = no; 2 = occasionally; 3 = sometimes; 4 = often
My personal hygiene habits are not different from those of local citizens	1 = completely disagree; 2 = disagree; 3 = basic agree; 4 = complete agree
Following the customs of my hometown is not important to me	1 = completely disagree; 2 = disagree; 3 = basic agree; 4 = complete agree

#### 2.2.3 Covariates

In recent years, scholars have focused on the utilization of public health services by migrants. Extensive research has demonstrated that sociodemographic characteristics such as age, gender, educational status, marital status, and household registration are linked to the utilization of health services. The household registration policy, enacted by the Chinese authorities in 1958, divides household registration by provinces and is categorized as either rural or urban. The household registration system allows individuals to receive social benefits in the area of their household registration, but also restricts access to social benefits outside the household registration. Individuals’ household registration is not fixed, as they can obtain a household registration in their place of residence through work or education.

Additionally, factors related to migration are also believed to influence the usage of public health services (22). Drawing upon existing studies and questionnaires, we chose six sociodemographic characteristics and four migration-related characteristics as covariates. The sociodemographic characteristics included gender (female or male), age (60–70, 70–80, above 80), education (primary school and below, junior middle school, senior middle school, college degree or above), household registration (rural or urban), marital status (married or single), and health condition (poor health or good health). The migration-related characteristics include whether the individual migrated alone (yes or no), whether they have settled down (yes or no), and the scope of migration (inter-provincial, intra-provincial, or intra-city across counties), flow time(less than 1 year, 2–5 years, 6–9 years, more than 10 years).

### 2.3 Statistical analysis

Latent class analysis(LCA) was performed using Mplus7.4. LCA is a method used to account for the diversity within a population in observational data by identifying potential subgroups of individuals. This allows for the examination of how older adult migrants utilize public health services based on different characteristics within the population. The most suitable model is chosen by assessing various fit indices, such as the Akaike information criterion (AIC), Bayesian information criterion (BIC), sample size adjusted BIC (SSA-BIC), Lo–Mendell–Rubin likelihood ratio test (LMR-LRT), and bootstrap likelihood ratio test (BLRT). Lower values of AIC, BIC, and SSA-BIC indicate a better fit, with BIC being the most reliable criterion. The significance of *p*-values for LMR-LRT and BLRT results is important (*p* < 0.05) in determining the validity of the model. Additionally, the interpretability of the model should also be taken into account.

Data were analyzed using SPSS 21. The dimensions of social integration were analyzed using exploratory factor analysis (27). Categorical variables were described with frequencies and percentages. Continuous variables were presented with means with standard deviations (SD). Chi-square tests were used to examine between-group differences for categorical variables, while ANOVA was used for the comparison among continuous variables, followed by *post hoc* analyses, and the significance levels were adjusted using the Tukey HSD method. Finally, multivariate logistic regression was used to investigate the factors influencing the potential categories of public health service utilization among the older mobile population.

## 3 Results

### 3.1 Basic information of the older adult floating population

The study consisted of 6,187 older adult migrants, with an average age of 66.09 ± 5.62 years. The majority of participants (77.8%) fell within the 60 to 70 age range. Out of the total sample, 3,581 participants were male (57.9%), while 2,606 were female (42.1%). A significant portion (47.8%) of the participants had completed only primary school or below, amounting to 2,955 individuals. Furthermore, 3,553 participants held a rural household registration, accounting for 57.4% of the population. The majority of participants (84.1%) were married, amounting to 5,204 individuals, while 1,497 individuals were solo migrants (24.2%). Approximately 44% of the older adult floating population expressed a desire to settle in their current location. In terms of self-reported health status, the majority (81.2%) described themselves as healthy or fairly healthy. Regarding migration patterns, 2,734 participants migrated between provinces (44.2%), while 34.8% moved within cities and 21% moved between counties. Only 9% of older adult migrants have been flown for less than a year. Totaling 2,339 older adult migrants, have been flown for over 10 years, making up 37.8% of the total. Descriptive statistics for these variables are presented in Table 2.

**Table 2:** Public health service subgroups with different demographic characteristics.

Characteristics		Overall situation	Class 1 (*n* = 3,264)	Class 2 (*n* = 1,743)	Class 3 (*n* = 1,180)	**χ** ^**2**^	*p*
Gender	Male	3,581 (57.9%)	1,833 (56.2%)	1,039 (59.6%)	709 (60.1%)	8.462	0.015
	Female	2,606 (42.1%)	1,431 (43.8%)	704 (40.4%)	471 (39.9%)		
Age	60–70	4,814 (77.8%)	2,503 (76.7%)	1,358 (77.9%)	953 (80.8%)	16.146	0.003
	70–80	1,173 (19.0%)	638 (19.5%)	327 (18.8%)	208 (17.6%)		
	Above 80	200 (3.2%)	123 (3.8%)	58 (3.3%)	19 (1.6%)		
Education	Primary school and below	2,955 (47.8%)	1,635 (50.1%)	843 (48.4%)	477 (40.4%)	41.642	0.000
	Junior middle school	1,867 (30.2%)	934 (28.6%)	550 (31.6%)	383 (32.5%)		
	Senior middle school	967 (15.6%)	490 (15.0%)	255 (14.6%)	222 (18.8%)		
	College degree or above	398 (6.4%)	205 (6.3%)	95 (5.5%)	98 (8.3%)		
Household registration	Rural	3,553 (57.4%)	1939 (59.4%)	1,013 (58.1%)	601 (50.9%)	25.927	0.000
	Urban	2,634 (42.6%)	1,325 (40.6%)	730 (41.9%)	579 (49.1%)		
Marital status	Married	5,204 (84.1%)	2,722 (83.4%)	1,472 (84.5%)	1,010 (85.6%)	3.345	0.188
	Single	983 (15.9%)	542 (16.6%)	271 (15.5%)	170 (14.4%)		
Whether to flow alone	Yes	1,497 (24.2%)	757 (23.2%)	421 (24.2%)	319 (27.0%)	6.975	0.003
	No	4,690 (75.8%)	2,507 (76.8%)	1,322 (75.8%)	861 (73.0%)		
Whether to settle down	Yes	2,722 (44.0%)	1,449 (44.4%)	736 (42.2%)	537 (45.5%)	3.952	0.413
	No	2,249 (36.4%)	1,186 (36.3%)	652 (37.4%)	411 (34.8%)		
	Not clear	1,216 (19.7%)	629 (19.3%)	355 (20.4%)	232 (19.7%)		
Health condition	Health	5,021 (81.2%)	2,568 (78.7%)	1,435 (82.3%)	1,018 (86.3%)	34.877	0.000
	Ill health	1,166 (18.8%)	696 (21.2%)	308 (17.7%)	162 (13.7%)		
Flow range	Trans-provincial	2,734 (44.2%)	1,558 (47.7%)	706 (40.5%)	470 (39.8%)	39.645	0.000
	Inter-city within the province	2,156 (34.8%)	1,092 (33.5%)	640 (36.7%)	424 (35.9%)		
	Within the city across the county	1,297 (21.0%)	614 (18.8%)	397 (22.8%)	286 (24.2%)		
Flow time (year)	≤1	559 (9.0%)	301 (9.2%)	164 (9.4%)	94 (8.0%)	15.573	0.016
	2–5	1903 (30.8%)	988 (30.3%)	528 (30.3%)	387 (32.8%)		
	6–9	1,386 (22.4%)	699 (21.4%)	389 (22.3%)	298 (25.3%)		
	≥10	2,339 (37.8%)	1,276 (39.1%)	662 (38.0%)	401 (34.0%)		

### 3.2 Results of LCA of public health services

As shown in Table 3, this study adopted five models to conduct LCA. The AIC and BIC values showed a gradual decrease across the models, whereas both LRT and BLRT values were found to be statistically significant in all five models. Additionally, the entropy surpassed 0.8 for each of the models. Based on the proportions of different categories in the table, it becomes apparent that if there are more than three categories and the potential categories account for less than 10%, a substantial disparity would exist between the samples. It has the potential to introduce inaccuracies into the model. Therefore, considering the actual situation and the accuracy of the results, it is advisable to opt for the most concise model, namely the third model, which classifies the utilization of public health services by the older adult floating population into three potential categories.

**Table 3:** Model fit statistics for latent class analysis models specifying one to five classes.

Model	AIC	BIC	SSA-BIC	Entropy	LMR-LRT	BLRT	Class size (%)
1	74597.3	74664.6	74632.8	–	–	–	–
2	52676.4	52817.8	52751.0	0.932	<0.00001	<0.00001	31.6/68.4
**3**	**49443.5**	**49658.9**	**49557.2**	**0.876**	**<0.00001**	**<0.00001**	**52.8/28.2/19**
4	49012.8	49302.2	49165.6	0.880	<0.00001	<0.00001	22/18.6/6.4/53
5	48600.3	48963.7	48792.1	0.830	<0.00001	<0.00001	7.4/16.4/19.3/7/50

AIC, Akaike Information Criteria; BIC, Bayesian Information Criteria; SSA-BIC, Adjusted Bayesian Information Criteria; LMR-LRT, LoMendell-Rubin Likelihood Ratio; BLRT, Bootstrapped Likelihood Ratio Tests. Smaller numbers for AIC, BIC, and SSA-BIC metrics represent a better model fit.The *p*-value of LMR-LRT, BLRT is significant then it means that the fit of model K is better than that of model K−1. Bolded row represents the selected model.

**Figure 1:**
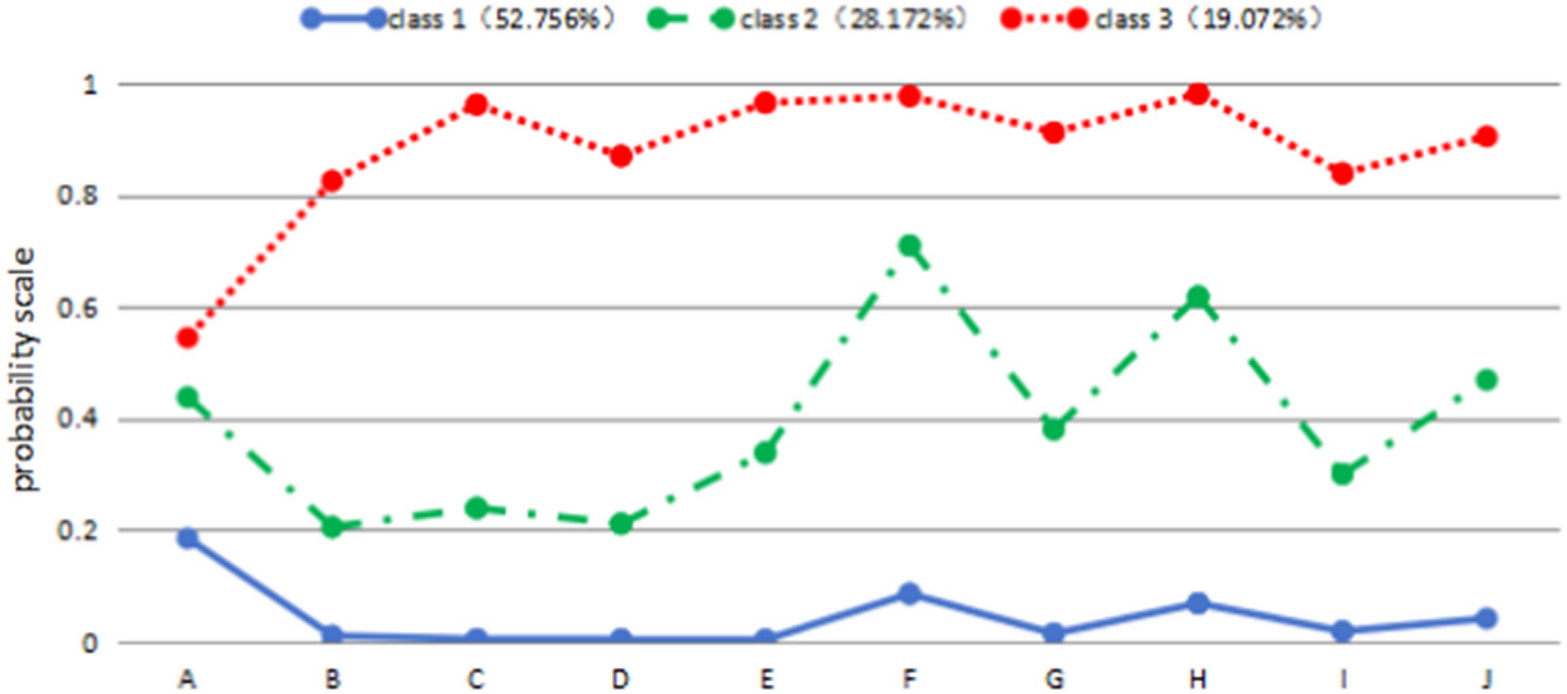
Distribution of characteristics of three potential subgroups of public health service utilization among older mobile populations. (A) Health records; (B) occupational disease; (C) sexually transmitted diseases; (D) reproductive health; (E) tuberculosis control; (F) smoking education; (G) mental health; (H) chronic disease; (I) maternal and child health; (J) public emergencies.

Class 1, named “low utilization of basic public health services class,” had the largest sample size of 3,264 cases (52.756%). The majority of subjects in this class did not have established health records and did not receive any health education. However, a few individuals had received education on smoking cessation and the prevention and treatment of chronic diseases. Class 2, named “medium utilization of basic public health services class,” consisted of 1,743 patients (28.172%). This class had intermediate levels of established health records and received some health education. Class 3, named “high utilization of basic public health services class,” had the smallest sample size of 1,180 cases (19.072%). This class had the best-established health records and the highest level of health education. These classes were identified based on the patterns observed in the data, and their distribution can be visualized in Figure 1.

### 3.3 Differences of social integration in the utilization of public health services

KMO and Bartlett’s test of sphericity for the selected indicators of social integration showed that they were suitable for factor analysis (KMO = 0.63; Bartlett’s test of sphericity **χ**^**2**^ = 13391.969, *p* < 0.001). Based on the outcomes of the exploratory factor analysis, four common factors were derived and designated as psychological integration, economic integration, social participation integration, and cultural integration (Table 4). Psychological integration among older adult migrants refers to their inner thoughts and feelings about integrating into their new place of residence. This can be measured by their expressions of affection and concern for the new location, as well as their subjective willingness to become a part of the local community. Economic integration is assessed through indicators such as monthly income and expenditure, providing insight into the financial situation and spending habits of older adult migrants. Social participation integration examines the extent to which older adult migrants engage in social activities within their new communities, focusing on their involvement in community management and provision of policy advice to the government. Cultural integration assesses the degree to which older adult migrants have embraced the cultural norms and practices of their new environment, particularly in terms of personal hygiene and attitudes towards local customs. The scores corresponding to each common factor, as well as the total score for social integration, were computed based on the assigned scores. A higher score reflected a better overall level of social integration. The older adult floating population showed an average social integration score of 22.04 ± 2.741, with the highest score of 3.49 ± 0.514 in psychological integration. It was followed by economic integration with the score of 2.42 ± 0.929, cultural integration with the score of 2.26 ± 0.688, and the lowest score of 1.10 ± 0.342 was observed in the social participation integration dimension. The ANOVA results indicated a significant difference in social integration levels among the three potential classes of public health service utilization. *Post hoc* comparisons revealed that the class with high utilization of public health services obtained notably higher scores in psychological integration and social participation compared to those in class 1 and class 2. Additionally, class 2 scored higher than class 1 in these domains. In terms of economic integration, although no significant difference was found between class 1 and class 2, class 3 scored significantly higher than both. Class 1and class 2 had significantly higher scores in cultural integration compared to class 3. Specific scores are shown in Table 5.

**Table 4:** Post-rotation social integration factor components.

Item	Factor			
	Psychological integration	Economic integration	Social participation integration	Cultural integration
The degree of inflow of liking	**0.871**	0.004	0.008	−0.079
The degree of inflow of concern	**0.858**	0.015	0.046	−0.042
The willingness to merge into the ground	**0.848**	0.004	0.014	−0.092
Monthly income	−0.012	**0.913**	0.047	−0.025
Monthly expenditure	0.030	**0.912**	0.029	−0.32
Willingness to participate in public affairs	0.023	0.021	**0.867**	0.012
Provide policy advice	0.031	0.052	**0.864**	−0.024
Health habit	0.000	0.028	−0.019	**0.831**
Social customs	−0.169	−0.082	0.008	**0.763**
Eigenvalues	2.244	1.674	1.504	1.291
% of variance explained	24.933	18.597	16.712	14.345
% of total variance explained	74.588			

Bold values in the same column indicate that the items are categorized in the same factor.

**Table 5:** Social integration score of the older mobile population and implications for public health service subgroup.

	Class 1 (*n* = 3,264)	Class 2 (*n* = 1,743)	Class 3 (*n* = 1,180)	F	*p*	Tukey HSD
Psychological integration	3.44 ± 0.516	3.51 ± 0.504	3.60 ± 0.507	38.774	0.000	1<2 2<3 1<3
Economic integration	2.40 ± 0.951	2.39 ± 0.923	2.51 ± 0.864	7.120	0.001	1<3 2<3
Social participation integration	1.06 ± 0.272	1.12 ± 0.361	1.18 ± 0.453	55.582	0.000	1<2 2<3 1<3
Cultural integration	2.30 ± 0.693	2.26 ± 0.665	2.18 ± 0.699	6.552	0.000	3<1 3<2
Total score	21.86 ± 2.735	22.04 ± 2.725	22.52 ± 2.724	189.563	0.000	1<3 2<3

### 3.4 Results of multivariate logistic regression

When examining the differences between class 1 and class 2 within the older migrant population, several findings emerged. Firstly, it was found that males (OR = 1.134, *p* = 0.001) and those with lower secondary education (OR = 1.332, *p* = 0.002) were more likely to belong to class 2. Conversely, migrants who moved across provinces were more inclined to be classified in class 1 (OR = 0.705, *p* < 0.001). Additionally, individuals with better psychological and social integration were more likely to be categorized into class 2 (OR = 1.222, *p* = 0.001; OR = 1.761, *p* < 0.001). In comparing class 1 and class 3, it was found that those who migrated alone were more likely to belong to class 3 (OR = 1.218, *p* < 0.001), while those who moved across provinces or cities were more likely to be classified in class 1 (OR = 0.582, *p* < 0.001; OR = 0.804, *p* < 0.001). Social integration also played a significant role in the categorization of class 1 and class 3. Individuals with better psychological, social participation, and economic integration had a higher likelihood of belonging to class 3 (OR = 1.685, *p* < 0.001; OR = 2.425, *p* < 0.001; OR = 1.086, *p* = 0.028), whereas those with better cultural integration were more likely to be excluded from class 3 (OR = 0.841, *p* < 0.001). Lastly, when comparing class 2 and class 3, individuals with better psychological, social participation, and economic integration were more likely to belong to class 3 rather than class 2 (OR = 1.369, *p* < 0.001; OR = 1.326, *p* = 0.002; OR = 1.092, *p* = 0.046). Detailed data are shown in the Table 6.

**Table 6:** Multiple logistic regression analysis of public health service utilization among the older adult mobile population.

	β	OR	95%CI	*p*
Class 2 vs. Class 1 (class 1 as a reference class)				
Gender: male	0.126	1.134	1.002–1.284	0.001
Education: junior high school	0.287	1.332	1.008–1.761	0.002
Flow range: across provinces	−0.350	0.705	0.601–0.827	0.000
Psychological integration	0.201	1.222	1.089–1.372	0.001
Social participation integration	0.566	1.761	1.455–2.133	0.000
Class 3 vs. class 1 (class 1 as a reference class)				
Flow alone: Yes	0.197	1.218	1.035–1.432	0.000
Flow range: across provinces	−0.541	0.582	0.484–0.700	0.000
Flow range: cross-city	−0.218	0.804	0.668–0.968	0.000
Psychological integration	0.522	1.685	1.464–1.939	0.000
Social participation integration	0.886	2.425	2.004–2.935	0.000
Economic integration	0.082	1.086	1.009–1.168	0.028
Cultural integration	−0.173	0.841	0.762–0.929	0.000
Class 3 vs. class 2 (class 2 as a reference class)				
Psychological integration	0.314	1.369	1.172–1.599	0.000
Social participation integration	0.282	1.326	1.108–1.586	0.002
Economic integration	0.008	1.092	1.001–1.191	0.046

## 4 Discussion

This study explored the connection between public health service utilization and social integration among the older adult migrant population, utilizing data from the 2017 CMDS. The results of LCA indicated that most of the population had inadequate access to public health services, categorized as class 1 and class 2. Less than 20 % belonged to class 3. These results underscore the suboptimal utilization of public health services among the older adult population (28). Specifically, class 1 demonstrated poor overall utilization of public health services, excluding health record establishment and education on smoking and chronic diseases. This could be attributed to their limited health awareness and hesitance to participate in health education and public health services. Their willingness to engage in education on chronic diseases and smoking, however, suggests a selective acceptance of health education within this class. In comparison, class 2 showed a moderate level of acceptance towards public health services compared to the other two classes. They possessed sound knowledge of public health services, demonstrated high health awareness, and actively engaged in such services. However, they showed less involvement in the prevention and treatment of occupational and sexually transmitted diseases, as well as reproductive health and maternal and child care. They had a preference for health education on smoking and chronic diseases. Additionally, both class 1 and class 2 displayed a higher demand for knowledge regarding smoking and chronic diseases. Therefore, it is recommended that relevant departments provide targeted health education to address the specific needs of the older adult migrant population. On the other hand, class 3 demonstrated the highest level of engagement in public health services, exhibiting a strong interest in various health education programs. Although their participation in health records was not significantly different from class 1 and class 2, this can be attributed to government policies and oversight in this domain. Acceptance of health education is largely driven by individual willingness, with those who are more health-conscious being more likely to engage in such programs. In contrast, the establishment of health records is overseen by government and healthcare institutions, making individual health awareness less influential in this regard.

The complex nature of social integration among the floating population can be examined from various perspectives. In this study, social integration was specifically divided into four dimensions: psychological integration (level of attachment, level of concern, willingness to assimilate), economic integration (income, expenses), social participation integration (willingness to engage in public affairs, providing policy recommendations), and cultural integration (health practices, societal norms). The results demonstrated that the older adult floating population displayed the highest levels of psychological integration, indicating a strong emotional connection and a desire to adapt to their current location. However, in addition to social participation integration, which scored the lowest, older people’s cultural integration scores were also at the lower end of the scale. This suggests difficulties in embracing the customs, traditions, and health behaviors of their new environment. On one hand, their aspiration for a safe and happy life drove their strong inclination towards psychological integration. On the other hand, their traditional values of honoring the older adult and cherishing their hometown influence their adherence to the customs and cultural norms of their hometown even in their new residence (29, 30).

Based on the findings regarding the impact of social integration on the utilization of public health services, it could be concluded that each dimension significantly influenced the utilization of such services. Notably, improved cultural integration among the older adult floating population was associated with a decreased utilization of public health services, unlike the other dimensions. This association might be attributed to educational background, which exerted a significant impact on both the cultural integration of older migrants and their utilization of public health services. Older individuals with higher levels of education may possess a strong self-awareness that hindered their cultural integration within the community (31). On the other hand, individuals with lower levels of education may find it easier to adapt to the local culture but may lack awareness of medical facilities and national policies, leading to suboptimal utilization of public health services. The utilization of public health services by the older adult floating population is positively influenced by the other three dimensions and overall social integration. Previous studies have shown that embracing public health services can improve social integration (32), indicating a mutually reinforcing relationship between social integration and public health services. Enhancing social integration can improve the access to public health services for the older adult floating population, which in turn can enhance their sense of identity and social integration. It is crucial to leverage this connection to continuously enhance relevant support measures and improve the social integration of the older adult floating population while also enhancing public health services.

The utilization of public health services among older adult migrants is influenced by various factors, as evidenced by multivariate analysis. Notably, senior males with at least a junior high school education are more likely to belong to class 2 rather than the class 1. This suggests that higher levels of education can influence access to healthcare services for older adult male migrants. In a previous study, it was observed that women in the migrant population were more receptive to health education compared to men (33). However, the present study revealed an opposite trend. This difference could be attributed to varying health education needs between younger and older individuals. Prior research has demonstrated that women prioritize maternal and child healthcare, eugenics, and contraception in health education acceptance, while older individuals prioritize prevention and treatment of chronic diseases. It has been suggested that the reduced emphasis on maternal and child healthcare among older women may contribute to their lower acceptance of health education (34). Similarly, older adult individuals who relocate alone are more likely to belong to the higher class. This could be attributed to the fact that older individuals who move without family or friends in their new area tend to connect more with the local community over time. Consequently, they become more familiar with local public health resources, facilitating their access to public health services. This underscores the positive impact of social networks and integration within one’s place of residence on the utilization of public health services. Conversely, older adult individuals who move to different municipalities or provinces often encounter challenges in accessing public health services due to increased mobility, significant changes in living and cultural environments (35), and potential language barriers (36). Their lack of familiarity with local services hinders their effective utilization of healthcare resources, making integration into the local community and accessing public health services more difficult. Overall, these findings emphasize the significance of education, social integration, and familiarity with local resources in determining the utilization of public health services among older adult migrants. Addressing these factors can help improve access and overall health outcomes for this vulnerable population.

It is important to acknowledge several limitations of the study. Firstly, the cross-sectional design used in this study limited the ability to establish causal relationships. Future research employing longitudinal designs would be valuable in exploring the stability of the identified classes over time. Secondly, relying on participants’ recollection for reporting their use of public health services introduced the possibility of recall bias, potentially affecting the accuracy of the data. The use of objective measures, such as medical records or administrative data, could enhance the reliability of the findings. Furthermore, the subjective nature of the criteria used to measure social integration might introduce bias and affect the fairness and accuracy of the results. Future studies could incorporate validated measures or employ multiple indicators to provide a more comprehensive assessment of social integration. In addition, due to the limitations of the database itself, many variables that may have an impact on the utilization of public health services by the older adult migrant population were not included in this study. For example, the lifestyle of the older adult as well as their medical habits may have a direct impact on their utilization of public health services, and it is suggested that future studies may explore these variables. It is also important to recognize that the study focused specifically on the older adult migrant population in China. Therefore, it remains unclear whether these findings can be generalized to other demographic groups or populations. Future research should address these limitations and explore various subgroups within the migrant population to gain a more comprehensive understanding of social integration and public health service utilization.

## 5 Conclusion

The study identified three distinct classes of public health service utilization among the older adult mobile population: low, medium, and high. The high utilization class was more likely to include older migrants who were male, had higher education, moved alone, and had a relatively short distance of migration. Furthermore, the findings indicated that good social integration was associated with increased utilization of public health services, whereas cultural integration seemed to hinder access to these services. To enhance the utilization of public health services among older adult migrants, it is crucial for relevant authorities to tailor these services to meet the specific needs of this population. A key strategy would be to prioritize the promotion of social integration among older adult migrants.

## Data availability statement

Publicly available datasets were analyzed in this study. This data can be found at: https://www.chinaldrk.org.cn/wjw/#/data/classify/population/yearList.

## Author contributions

XB: Writing – original draft, Writing – review & editing, Conceptualization, Data curation, Software. TW: Conceptualization, Data curation, Writing – review & editing, Writing – original draft. QD: Conceptualization, Software, Writing – review & editing, Writing – original draft. CL: Conceptualization, Software, Supervision, Writing – review & editing, Writing – original draft.
